# Silver Nanoclusters Tunable Visible Emission and Energy Transfer to Yb^3+^ Ions in Co-Doped GeO_2_-PbO Glasses for Photonic Applications

**DOI:** 10.3390/nano13071177

**Published:** 2023-03-25

**Authors:** Augusto Anselmo Amaro, Guilherme Rodrigues da Silva Mattos, Marcos Vinicius de Morais Nishimura, Jessica Dipold, Niklaus Ursus Wetter, Luciana Reyes Pires Kassab

**Affiliations:** 1 Departamento de Engenharia de Sistemas Eletronicos, Escola Politécnica da Universidade de São Paulo, Av. Prof. Luciano Gualberto, 158, Travessa 3, São Paulo 05508-900, SP, Brazil; augustoaamaro@usp.br (A.A.A.);; 2 Instituto de Pesquisas Energéticas e Nucleares, IPEN-CNEN, 2242, Av. Prof. Lineu Prestes, São Paulo 05508-000, SP, Brazil; 3 Faculdade de Tecnologia de São Paulo, CEETEPS, Praça Cel. Fernando Prestes, 30, São Paulo 01124-060, SP, Brazil

**Keywords:** silver nanoclusters, ytterbium ions, germanate glasses, tunable luminescence

## Abstract

This work investigates the optical properties of Yb^3+^ ions doped GeO_2_-PbO glasses containing Ag nanoclusters (NCs), produced by the melt-quenching technique. The lack in the literature regarding the energy transfer (ET) between these species in these glasses motivated the present work. Tunable visible emission occurs from blue to orange depending on the Yb^3+^ concentration which affects the size of the Ag NCs, as observed by transmission electron microscopy. The ET mechanism from Ag NCs to Yb^3+^ ions (^2^F_7/2_ → ^2^F_5/2_) was attributed to the S_1_→T_1_ decay (spin-forbidden electronic transition between singlet–triplet states) and was corroborated by fast and slow lifetime decrease (at 550 nm) of Ag NCs and photoluminescence (PL) growth at 980 nm, for excitations at 355 and 405 nm. The sample with the highest Yb^3+^ concentration exhibits the highest PL growth under 355 nm excitation, whereas at 410 nm it is the sample with the lowest concentration. The restriction of Yb^3+^ ions to the growth of NCs is responsible for these effects. Thus, higher Yb^3+^ concentration forms smaller Ag NCs, whose excitation at 355 nm leads to more efficient ET to Yb^3+^ ions compared to 410 nm. These findings have potential applications in the visible to near-infrared regions, such as tunable CW laser sources and photovoltaic devices.

## 1. Introduction

Several reports have pointed out important photonic applications of silver (Ag) nanoclusters (NCs) in undoped and rare-earth doped glasses prepared by the melt quenching technique [1]. Most of the reports about Ag NCs were based on liquid, polymer, or organic materials [2]. Only more recently, the possibility of using oxyfluoride glasses [3] was demonstrated as they have advantages for the production of fibers, films, and other forms when compared to other materials [3,4]. Since then various glassy compositions have been reported in the literature as potential materials to host Ag NCs, such as fluorophosphates [5,6,7], zinc borate [8] CABAl [9], oxyfluorides [10,11], borosilicates [12,13], and others. The corresponding applications presented in these reports include solar cells, flexible screen monitors, white light generation, tunable visible light emission sources, and CW lasers. Ag NCs consist of the accumulation of a few tens of Ag atoms and Ag^+^ free ions [2,6]; they are amorphous, different to the metallic nanoparticles (NPs) [14] that are crystalline and have larger dimensions. Over tens of Ag atoms and Ag NCs aggregates form the metallic NPs evidenced by the absorption plasmon band [5]. Ag NCs differ from metallic NPs in the discretization of energy levels and, therefore, exhibit photoluminescence (PL), which depends on the laser excitation wavelength as well as on the glass composition and Ag concentration. The adequate concentration of Ag NCs also plays an important role; high concentration will facilitate their agglomeration and the formation of metallic NPs [5]. Ag NCs have special optical properties that are different from the bulk of Ag and single Ag atoms. The former is normally not luminescent, whereas the latter exhibit a narrow PL band in the UV–blue region of the electromagnetic spectrum [15]. On the other hand, Ag NCs normally exhibit a broad band that covers the visible range of the electromagnetic spectrum [2,3,4,15]. The PL is also correlated with the size of Ag NCs and can be tuned from the UV to visible (VIS) and near-infrared (NIR) by the choice of an adequate excitation wavelength. Both Ag NCs and Ag NPs have been demonstrated to be sensitizers for lanthanides, as they are capable of increasing their PL. Ag NPs enhance the PL of the rare-earth ions by the localized surface plasmon resonance (coherent oscillation of free electrons at a specific frequency that produces the enhanced local field in the vicinities of the rare-earth ions) [4,14,16,17,18,19]. On the other hand, the Ag NCs do not have these plasmonic properties and the energy transfer (ET) is the only possible mechanism that has been demonstrated [8,12,20,21,22]. In some cases, when the PL bands of Ag NCs and those of rare-earth ions are mixed, white light generation and tunable visible light can take place depending on the excitation wavelength. Potential applications of rare-earth ions doped GeO_2_-PbO glasses with metallic NPs were reported in the literature as waveguides to operate in the infrared region based on fs laser irradiation [23] and on the pedestal architecture [24] cover layer to enhance Si solar cell efficiency [25] and devices to promote white light emission [26]. However, GeO_2_-based glasses with Ag NCs have rarely been investigated, which motivated the present study. These glasses have important characteristics for photonics, such as low cut-off phonon energy (800 cm^−1^), which is relevant for reducing non-radiative losses, high refractive index (~2.0), large nonlinear optical properties, and wide transmission window (400–5000 nm). The viscosity of these materials depends on the annealing temperature. The growth of Ag NCs is favored in media with high viscosity, which can be achieved by annealing temperatures below the glass transition, as shown for fluorophosphate glasses [5]. Motivated by these results, we developed a way to grow Ag NCs in GeO_2_-PbO glasses by using annealing temperatures of 400 °C, below the glass transition temperature [27]; we also reported that higher annealing temperatures (470 °C) decrease the material’s viscosity, favoring Ag NCs aggregation and, consequently, the production of Ag NPs [27]. This work investigates the optical properties of Yb^3+^ doped GeO_2_-PbO glasses with Ag NCs; absorbance, PL, and transmission electron microscopy results are presented. The PL lifetime decay results of Ag NCs (VIS region) and Yb^3+^ ions (NIR region) are shown to explain the ET mechanism between both species. Visible tunable light emission is also discussed based on the influence of Yb^3+^ ions that limit the Ag NCs size distribution [3]. The ET processes between Ag NCs and Yb^3+^ ions have been reported for different glasses [3,12,28,29]. The present investigation shows for the first time the potential of GeO_2_-PbO glasses to host Yb^3+^ ions and Ag NCs as well as the ET mechanisms between them. When optically excited in the UV–blue region, PL is observed in both, the VIS region due to Ag NCs, and in the NIR region due to the Yb^3+^ ions, with potential for applications for very broad band light sources, lasers, and down-conversion mechanisms, which can be used to enhance photovoltaic device performance, as reported in [28,29]. The present results contribute to the researchers who work in this field and also open up new perspectives for improving the optical properties of different rare-earth ions by the presence of Ag NCs.

## 2. Materials and Methods

Samples of lead-germanate glass with composition (in wt%) of 40.0% GeO_2_ and 60.0% PbO (GP) were constructed using the well-known melt-quenching glass fabrication technique. The base composition was doped with different concentrations of AgNO_3_ and Yb_2_O_3_ (4.5% AgNO_3_, 4.5% AgNO_3_/2.0% Yb_2_O_3_, 4.5% AgNO_3_/4.5% Yb_2_O_3_, 2.0% Yb_2_O_3_, and 4.5% Yb_2_O_3_). The samples were, respectively, labelled as follows: GP 4.5Ag, GP 4.5Ag2Yb, GP 4.5Ag4.5Yb, GP 2Yb, and GP 4.5Yb. The reagents were melted in an alumina crucible at 1200 °C for one hour with mechanical stirring, then quickly poured on a pre-heated brass mold, and placed in a furnace at 420 °C for one hour to reduce the glass’s internal stresses. The sample was left inside the furnace and cooled down until the ambient temperature was reached. Samples with AgNO_3_ were annealed for 12 h to form Ag NCs. In oxyfluoride glasses, F^−^ favors the formation of Ag NCs color centers [3,30]. In GP glasses, the Ag^+^ ions nucleation takes place by matrix-assisted reduction enabled by the non-bridging oxygen which is the only source of negative charges available for this glass system [31,32]. Finally, the samples were polished and cut to be analyzed. To measure the absorption spectra in the VIS (400–800 nm) and NIR (900–1200 nm) regions, an Ocean Optics QE65 Pro spectrometer was used. Measurements of PL in the VIS region were carried out by a Varian Cary Eclipse Fluorescence Spectrometer. The PL decay lifetime of Ag NCs was measured to investigate the energy transfer from Ag NCs to Yb^3+^. During this analysis, the PL decay lifetime signals were recorded at the specific predetermined wavelengths after the excitation ceased. Ag NCs PL decay lifetime was fitted by a double exponential decay function, as shown in Equation (1) [6,15,22]:

(1) $$I\left(t\right)=A_{1}\;\cdot \;exp\;\left(\frac{-t}{\tau_{\mathrm{fast}}\;}\right)+A_{2}\;\cdot \;exp\;\left(\frac{-t}{\tau_{\mathrm{slow}}}\right)$$

The PL intensity is shown as “I”, $A_{1}$ and $A_{2}$ are constants, *t* is the lifetime and the τ_fast_ and τ_slow_ are the decay lifetimes related to spin-allowed (singlet–singlet and triplet–triplet) and spin-forbidden (singlet–triplet and triplet–singlet) electronic transitions, respectively, that will be explained in the next section. The NIR PL analysis was performed using an optical parametric oscillator (OPO) laser system (Opollete TM HE 335 LD model) from Opotek Inc. (Carlsbad, CA, USA), which was pumped by a Q-switched Nd:YAG laser. The OPO was tuned to excite the samples at 355 and 410 nm, and a filter was used to block the second harmonic of the laser at 710 and 820 nm, respectively. The emission signal was obtained in a perpendicular direction with respect to the incident beam. For the PL decay lifetime analysis of Yb^3+^ in the NIR region, a continuous-wave diode laser operating at 405 nm was used. To measure the lifetime, the sample’s emission signal was focused into a Newport Cornerstone 260 monochromator with an IR Ge detector directly connected to a Keysight DSO1024A oscilloscope. PL decay lifetimes were fitted by a single exponential decay function. The size and shape of NCs were investigated using transmission electron microscopes (TEM) with 200 and 300 kV; electron diffraction (ED) measurements were performed to corroborate the amorphous nature of Ag NCs. For these measurements, the samples were milled, mixed with distilled water, and partially decanted. The floating part was taken to an ultra-thin carbon film deposited on a Cu grid to be analyzed. All measurements were performed at room temperature.

## 3. Results and Discussion

**Figure 1:**
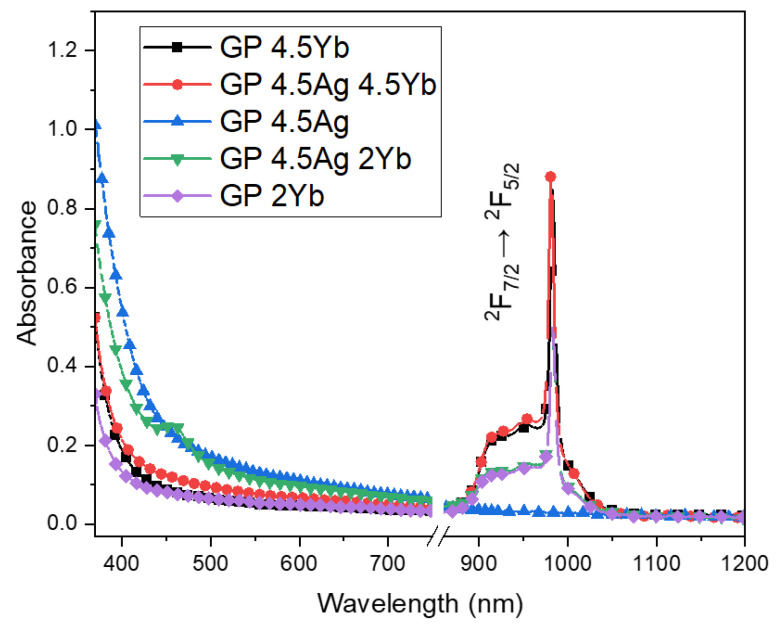
Absorption spectra of GP 2Yb, GP 4.5Yb, GP 4.5Ag, GP 4.5Ag2Yb, and GP 4.5Ag4.5Yb glass samples.

The absorption spectra of the GP glass samples doped with the specified concentrations of AgNO_3_ and Yb_2_O_3_ are displayed in Figure 1.

The GP 4.5Ag2Yb sample exhibits a plasmon absorption band, which is attributed to the formation of a small concentration of Ag NPs and not only NCs [6]. It is important to highlight that Ag NCs represent the accumulation of a few tens of Ag atoms and Ag^+^ free ions. This stage precedes the formation of Ag NPs, which are crystalline. Over tens of Ag atoms and Ag NCs aggregates form the metallic NPs evidenced by the absorption plasmon band [5]. When Yb^3+^ ions are added, they restrict the growth of the NCs and smaller NCs are formed [3]. So, when a larger concentration of Yb^3+^ is added, smaller NCs are formed. Therefore, the sample with the smallest concentration of Yb^3+^, which is the 4.5Ag2Yb sample, restricts less the Ag NCs growth and favors the formation of AgNPs; this explains the presence of the absorption plasmon band presented in Figure 1, for 4.5Ag2Yb sample. We cannot discard the formation of metallic NPs that may also exist but in small concentration, explaining the absence of the absorption plasmon band for 4.5Ag and 4.5Ag4.5Yb samples. Absorption bands associated with the (^2^F_7/2_ → ^2^F_5/2_) electronic transition of Yb^3+^ ions are also shown and increase with Yb^3+^ concentration.

**Figure 2:**
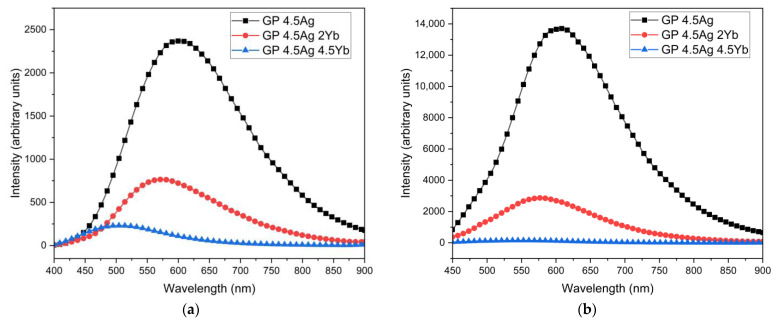
PL spectra of GP 4.5Ag, GP 4.5Ag2Yb, and GP 4.5Ag4.5Yb samples under excitation at (**a**) 355 nm and (**b**) 405 nm.

In Figure 2, the PL spectra of the samples are displayed. A broad band is observed in the VIS region, under excitations at 355 nm (Figure 2a) and 405 nm (Figure 2b).

**Figure 3:**
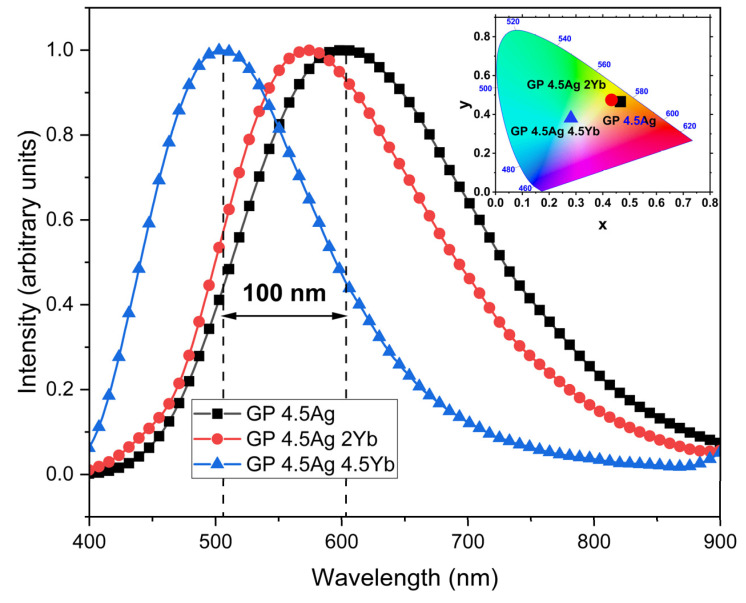
Normalized PL of GP 4.5Ag, GP 4.5Ag2Yb, and GP 4.5Ag4.5Yb samples under excitation at 355 nm. The inset shows the samples chromaticity diagram (CIE-1931).

The broad PL band (VIS-NIR) is attributed to the presence of Ag NCs with varying sizes, as will be explained. As the concentration of Yb^3+^ ions in the co-doped samples increases, the Ag NCs PL decreases for both excitation wavelengths. This reduction is attributed to the ET from the Ag NCs to the Yb^3+^ ions, as has been reported for other glass compositions [3,12,28,29]. Figure 3 shows the normalized PL spectra under 355 nm excitation; the corresponding chromaticity diagram (CIE-1931) is presented in the inset.

The emission peak of the Ag NCs is size-dependent: small Ag NCs emit in the blue region and large Ag NCs emit in the red region of the spectra [3,6]. The presence of Yb^3+^ ions restrict the size of the Ag NCs [3]; so, as the concentration of Yb^3+^ ions increases, smaller Ag NCs are formed, shifting the peak emission to the blue region. So, the results presented in the normalized spectra of Figure 3 demonstrate that Ag NCs emit varying colors and the redshift of about 100 nm indicates the presence of larger Ag NCs in the 4.5Ag2Yb sample when compared to the 4.5Ag4.5Yb sample. We highlight that tunable VIS light emission from blue to orange takes place, depending on Yb^3+^ ions concentration, as is demonstrated in the inset of Figure 3. As already explained, in GP glasses, the Ag^+^ ions nucleation takes place by matrix-assisted reduction due to the non-bridging oxygen, the only source of negative charges available. So, Ag^+^ and Yb^3+^ ions compete for reducing agents (non-bridging oxygens); consequently, the number of reducing agents available for Ag NCs is decreased and limits their sizes [31,32].

**Figure 4:**
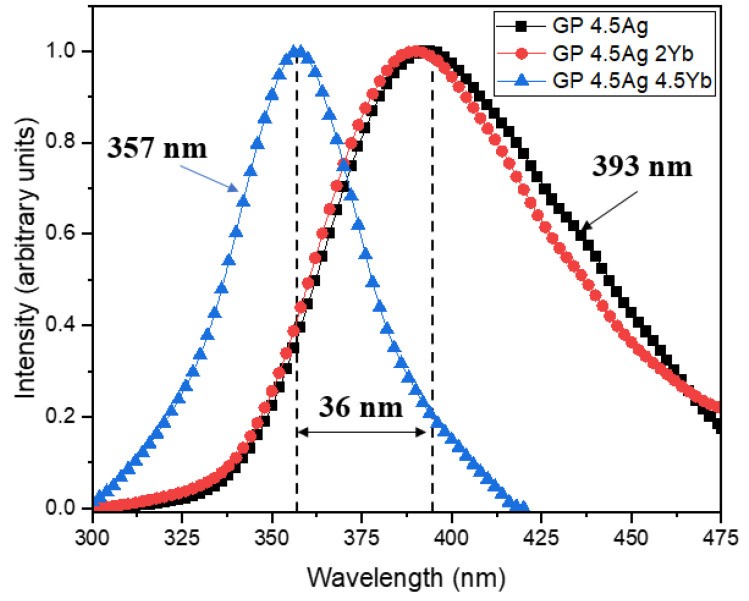
Ag NCs normalized excitation spectra (PLE) with PL fixed at 550 nm for GP 4.5Ag, GP 4.5Ag2Yb, and GP 4.5Ag4.5Yb.

Figure 4 shows the excitation spectra (PLE) of Ag NCs at 550 nm. We note that for the GP 4.5Ag sample, the best excitation wavelength for PL at 550 nm is around 393 nm, whereas, for a higher concentration of Yb^3+^, it is 357 nm. This is another indication that in samples with larger Yb_2_O_3_ concentrations smaller Ag NCs are formed [3].

**Figure 5:**
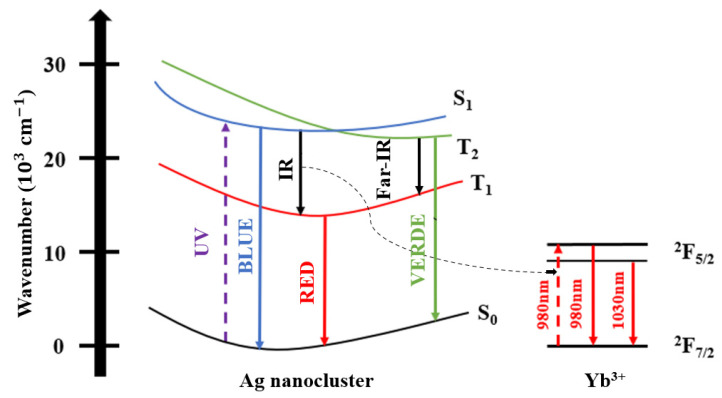
Simplified Ag NCs energy diagram with ET to Yb^3+^ ions.

The Ag NCs responsible for the absorption at shorter wavelengths are the small ones that have a high probability of singlet–singlet transitions. For a better understanding of the Ag NCs energy levels and ET to Yb^3+^ ions, Figure 5 presents a simplified energy diagram proposed by Vélazquez et al., based on experimental observations [15], where S_0_ represents the ground state, S_1_ the excited singlet state, T_1_ and T_2_ the excited triplet states, respectively. In this diagram, there are mainly five transitions for Ag NCs: emission in the blue region (S_1_ →S_0_), green/yellow region (T_2_ → S_0_), yellow/red region (T_1_ → S_0_), and IR and far-IR regions (S_1_ → T_1_ and T_2_ → T_1_, respectively). Spin-allowed electronic transitions are related to singlet–singlet (S_1_ → S_0_) and triplet–triplet (T_2_ → T_1_) states are related to spin-allowed electronic transitions whereas transitions between singlet–triplet (S_1_ → T_1_) and triplet–singlet (T_2_ → S_0_ and T_1_ → S_0_) states are considered spin-forbidden. So, when an excitation takes place at the UV region, a transition occurs from the ground state (S_0_) to the S_1_ excited state originating the blue emission by the spin-allowed transition (S_1_ → S_0_); moreover, relaxations to T_2_ and T_1_ triplet states originate the spin-forbidden T_2_ → S_0_ and T_1_ → S_0_ transitions, respectively, that in addition to S_1_ → S_0_ transition originate the broad band that covers the large region of the electromagnetic spectrum, as shown in Figure 2. According to Tikhomirov et al. [3,6], the region of the short wavelength of the PL spectrum corresponds to Ag NCs of smaller size, whereas the region of longer wavelength is associated to larger ones. Then the Ag NCs that emit in the blue region have the smallest size; as their size increases, the PL is redshifted [6]. Studies for other glass matrices have shown that ET from the Ag NCs to Yb^3+^ ions is probably due to large NCs, as shown by the black dotted arrow in Figure 5. Thus, it is the Ag NCs that emit in the NIR and transfer energy to nearby Yb^3+^ ions that are promoted to the excited ^2^F_5/2_ state [12,28,29]. The same energy diagram was used by Fares et al. [6] to explain the ET mechanism between Ag NCs and rare-earth ions.

Spin-allowed electronic transitions are related to singlet–singlet (S_1_ → S_0_) and triplet–triplet (T_2_ → T_1_) states, which present relatively short decay lifetimes. However, transitions between singlet–triplet (S_1_ → T_1_) and triplet–singlet (T_2_ → S_0_ e T_1_ → S_0_) states are considered spin-forbidden, being non-spontaneous and with long decay lifetimes [33,34].

Ag NCs PL around the UV-blue region is associated with fast decay times (τ_fast_) by spin-allowed transitions between energy states. On the other hand, Ag NCs PL in the green, yellow, red, and IR regions of the spectra display slow decay times (τ_slow_) and are associated with spin-forbidden transitions [6].

**Figure 6:**
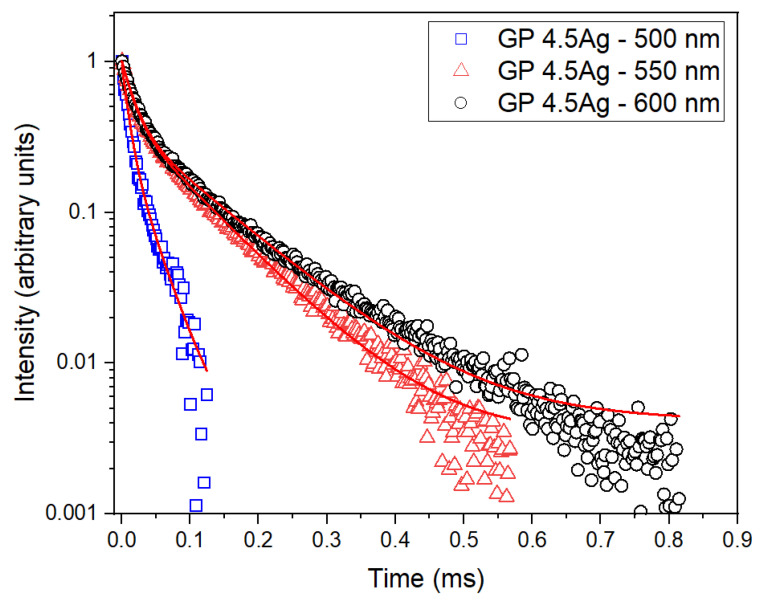
Ag NCs PL decay curves with detection at 500, 550, and 600 nm, under 405 nm excitation for the GP 4.5Ag sample.

The ET mechanism between Ag NCs and Yb^3+^ ions was studied. Ag NCs PL decay curves were measured for the GP 4.5Ag sample, under 405 nm excitation, with detection at 500, 550, and 600 nm as presented in Figure 6. Table 1 shows the lifetime values obtained by using Equation (1).

We observe that both τ_fast_ and τ_slow_ increase with wavelength. The highest τ_slow_ value is related to 600 nm detection that corresponds to the PL of large Ag NCs in the yellow/red region. This indicates that more Ag NCs spin-forbidden transitions take place at this detection wavelength. Also, PL lifetimes are shorter for emissions in the blue region due to the spin-allowed singlet–singlet transitions (S_1_ → S_0_) showing lower growth with the increase of the wavelength compared to the spin-forbidden transitions. The results presented in Table 1 corroborate the large size distribution of Ag NCs, as already observed in other glasses [5,6], and demonstrate the multiplicity of size distribution of Ag NCs that are responsible for the different lifetimes obtained.

**Figure 7:**
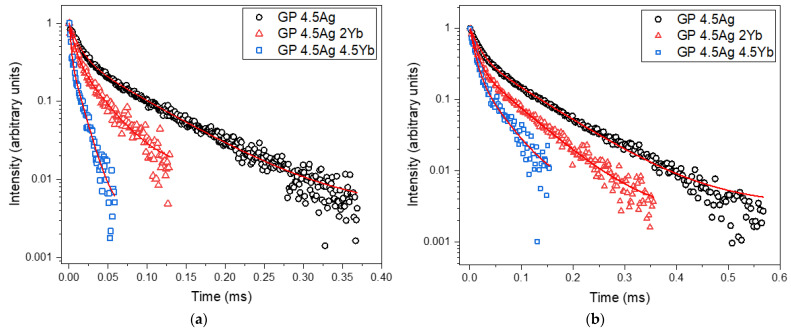
Ag NCs PL decay curves at 550 nm under (**a**) 355 nm and (**b**) 405 nm excitation for GP 4.5Ag, GP 4.5Ag2Yb, and GP 4.5Ag4.5Yb samples.

The PL decay curves at 550 nm, for Ag NCs under excitation at 355 nm and 405 nm are shown in Figure 7a,b, and the obtained lifetimes are listed in Table 2 and Table 3, respectively.

Table 2 and Table 3 demonstrate that both τ_fast_ and τ_slow_ decrease as Yb_2_O_3_ doping enhances, suggesting an efficient ET from Ag NCs to Yb^3+^ ions. These results are related to the PL results of the Ag NCs, presented in Figure 2, where the largest reduction takes place for the GP 4.5Ag4.5Yb sample, which has a larger concentration of acceptors Yb^3+^ ions (with respect to GP 4.5Ag2Yb sample) to receive ET, also leading to a larger reduction of the Ag NCs lifetime.

**Figure 8:**
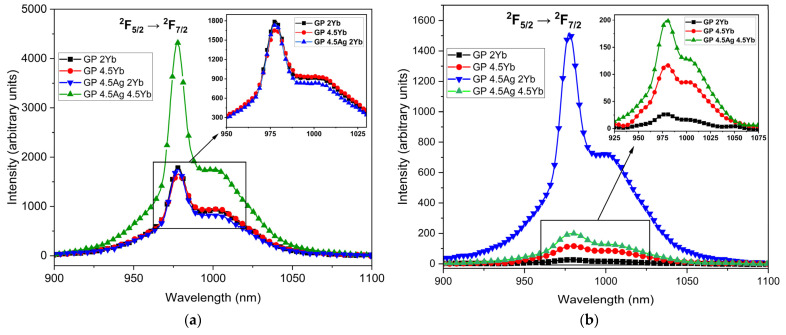
Yb^3+^ ions NIR luminescence for GP 2Yb, GP 4.5Yb, GP 4.5Ag2Yb, and GP 4.5Ag4.5Yb samples under (**a**) 355 nm and (**b**) 410 nm excitation.

NIR PL spectra of the Yb^3+^ ions at 980 nm are shown in Figure 8a,b under 355 nm and 410 nm excitations, respectively.

**Figure 9:**
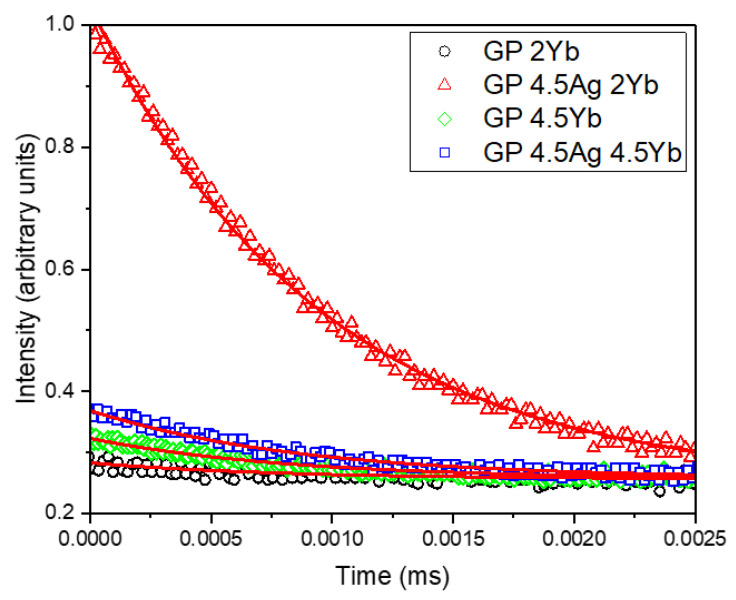
Yb^3+^ ions PL decay curves under 405 nm excitation with detection at 980 nm for GP 2Yb, GP 4.5Yb, GP 4.5Ag2Yb, and GP 4.5Ag4.5Yb samples.

A large PL enhancement at 980 nm (more than 100%) is observed for the GP 4.5Ag4.5Yb sample under 355 nm excitation. This sample also exhibited the lowest PL intensity at 550 nm, as shown in Figure 2a, suggesting that this is the adequate excitation wavelength to promote ET from Ag NCs to Yb^3+^ ions, due to the smaller Ag NCs that are best excited at 355 nm. Moreover, the GP 4.5Ag2Yb sample shows PL intensity similar to the samples without Ag NCs, indicating that there is a small number of Ag NCs with favorable dimensions to be excited under 355 nm and, consequently, provide ET to Yb^3+^ ions. On the other hand, under 410 nm excitation wavelength, the GP 4.5Ag2Yb sample showed the highest PL intensity at 980 nm associated with Yb^3+^ ions (more than 1000% with respect to the GP 2Yb sample), probably due to the larger Ag NCs that are best excited at longer wavelength (Figure 4). Moreover, the contribution of the surface plasmon resonance of Ag NPs (Figure 1), whose enhanced local field favors the PL of Yb^3+^ ions, at 980 nm, must be considered and also explains the higher PL of GP 4.5Ag2Yb sample when compared to GP 4.5Ag4.5Yb sample. These results can be attributed to the presence of Ag NCs with different sizes that influence the ET efficiency to Yb^3+^ ions. It is worth mentioning that GP samples without Ag NCs (GP 2Yb and GP 4.5Yb) also presented PL of Yb^3+^ ions under 355 and 410 nm excitations. This is probably caused by the excitation of Pb^2+^ ions, present in the GP glass matrix, which can transfer energy to the surrounding Yb^3+^ ions [35]. Similar behavior was observed for oxyfluoride glasses that showed ET to Yb^3+^ ions from the host [28,29]. Figure 9 displays the PL decay curves at 980 nm, associated with Yb^3+^ ions, under 405 nm diode laser excitation, whose lifetime values are shown in Table 4.

Comparing the results of GP 4.5Yb and GP 4.5Ag4.5Yb samples, we observe a slight lifetime increase (from 0.83 to 0.87 ms); on the other hand, for the GP 2Yb and GP 4.5Ag2Yb samples, a larger growth occurs (from 0.79 to 0.94 ms). The reduction of Ag NCs lifetime shown in Table 3, and the Yb^3+^ ions lifetime growth presented in Table 4, confirm the ET from Ag NCs to Yb^3+^ ions under 405 nm excitation. Although the GP 4.5Ag4.5Yb sample demonstrated the largest decrease for Ag NCs lifetimes, under 405 nm excitation (Table 3), that would suggest higher ET to this sample, it is the GP 4.5Ag2Yb sample that displayed the highest Yb^3+^ ions lifetime enhancement when excited at the same wavelength; this result indicates that the larger Ag NCs (Figure 3) formed in the GP 4.5Ag2Yb sample when compared to those present in GP 4.5Ag4.5Yb have more adequate sizes to be excited at 405 nm (Figure 4) and provide efficient ET to Yb^3+^ ions. The mentioned ET to the Yb^3+^ ions, as demonstrated in Figure 5, is due to the S_1_ → T_1_ transition of Ag NCs [12,28,29].

Finally, increased Yb^3+^ ions PL intensity at 980 nm was observed for excitations at 355 and 410 nm, respectively, depending on Yb^3+^ concentration; in the first case (355 nm), the largest increase occurs for the sample with higher Yb^3+^ concentration while, in the second case (410 nm), for the sample with a lower one. These findings can be attributed to the fact that Yb^3+^ restricts the growth of the Ag NCs [3].

**Figure 10:**
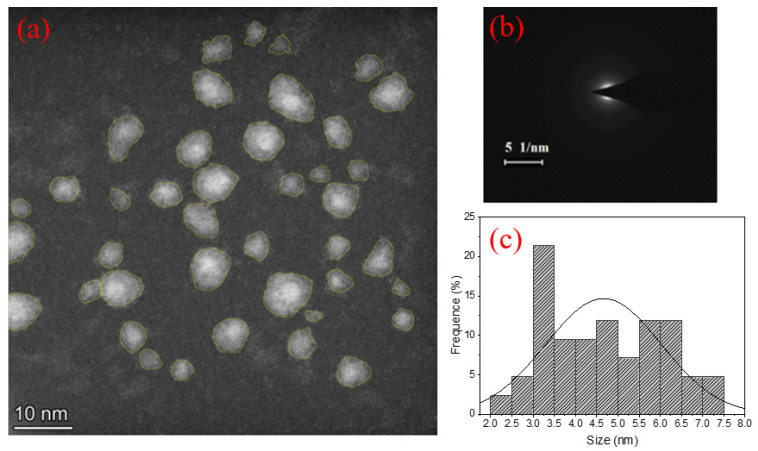
(**a**) TEM image of the GP 4.5Ag sample, (**b**) ED pattern, and (**c**) size distribution.

**Figure 11:**
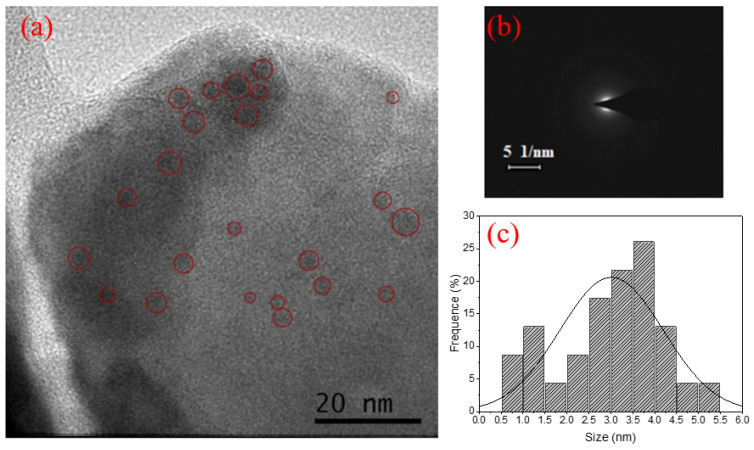
(**a**) TEM image of the GP 4.5Ag2Yb sample, (**b**) ED pattern, and (**c**) size distribution.

**Figure 12:**
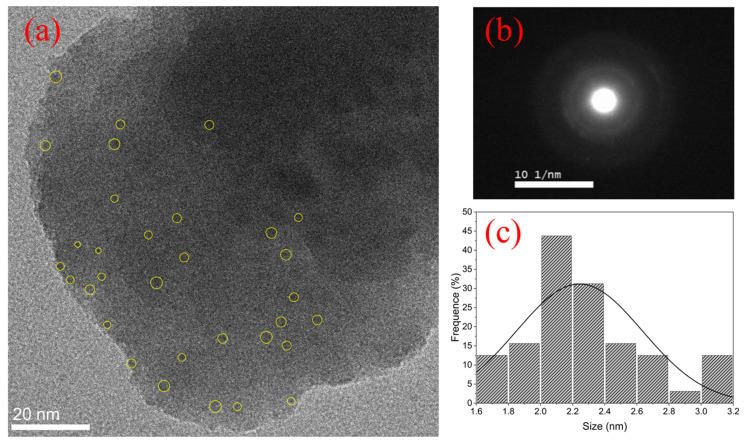
(**a**) TEM image of the GP 4.5Ag4.5Yb sample, (**b**) ED pattern, and (**c**) size distribution.

TEM images, ED patterns, and size distribution of Ag NCs in GP 4.5Ag, GP 4.5Ag2Yb, and GP 4.5Ag4.5Yb samples are shown in Figure 10, Figure 11 and Figure 12, respectively. The amorphous nature of Ag NCs is present in Figure 10b, Figure 11b and Figure 12b.

The influence of Ag NCs size restriction caused by Yb^3+^ ions [3] can be observed by comparing Figure 10c, Figure 11c and Figure 12c. The average sizes of the Ag NCs are 4.7 nm, 3 nm, and 2.2 nm for the GP 4.5Ag, GP 4.5Ag2Yb, and GP 4.5Ag4.5Yb samples, respectively. Furthermore, we observe that the maximum size of Ag NCs is 7.5 nm for the GP 4.5Ag sample, and 5.5 nm for the GP 4.5Ag2Yb sample, while for the GP 4.5Ag4.5Yb sample this value reduces to 3.2 nm. These results corroborate the effects discussed before regarding the PL, PLE, and lifetime measurements.

## 4. Conclusions

In this research, GeO_2_-PbO glasses doped with Yb^3+^ ions with and without Ag NCs were produced by the melt-quenching technique. The impact of Yb^3+^ on the size of Ag NCs was studied as well as its influence on the ET processes to Yb^3+^ ions. The emission peak of the Ag NCs is size-dependent: small Ag NCs emit in the blue region and large Ag NCs emit in the red region of the spectra. PL spectra showed a redshift of about 100 nm indicating the presence of larger Ag NCs in the 4.5Ag2Yb sample when compared to the 4.5Ag4.5Yb sample. This was attributed to the presence of Yb^3+^ ions that restricts the size of the Ag NCs; then tunable visible light emission occurs from blue to orange depending on the concentration of Yb^3+^ ions. Ag NCs PL decay curves were measured for the GP 4.5Ag sample, under 405 nm excitation, and the detection at 500, 550, and 600 nm corroborated the large size distribution of Ag NCs. Both τ_fast_ and τ_slow_ increase with wavelength; however, decay lifetimes are shorter for spin-allowed singlet–singlet transitions (S_1_ → S_0_) and also exhibit lower growth with wavelength compared to the spin-forbidden singlet–triplet (S_1_ → T_1_) and triplet–singlet (T_2_ → S_0_ and T_1_ → S_0_) transitions. The lifetime of Ag NCs showed a decrease (at 550 nm) in the presence of Yb^3+^ ions, for τ_fast_ and τ_slow_ components (at 355 and 405 nm excitation) and indicated a mechanism of ET to the Yb^3+^ ions attributed to the S_1_ → T_1_ decay (spin- forbidden transition between singlet–triplet states of Ag NCs). These results are related to the Ag NCs PL ones, in which the largest reduction takes place for the GP 4.5Ag4.5Yb sample, whose larger concentration of acceptors Yb^3+^ ions (with respect to GP 4.5Ag2Yb) to receive ET, led to a larger reduction of the Ag NCs lifetime. Additionally, the PL of Yb^3+^ ions indicated an Ag NCs size-dependent ET, based on the excitation wavelength. The sample with the largest Yb^3+^ ions concentration exhibits the highest PL growth at 980 nm (more than 100%), under 355 nm excitation. On the other hand, at 410 nm pump wavelength, it is the sample with the smallest concentration that presented PL enhancement of more than 1000%. The ability of Yb^3+^ ions to hamper the NCs growth is responsible for these effects. Larger Yb^3+^ ions concentration forms smaller Ag NCs, whose excitation at 355 nm leads to more efficient ET to Yb^3+^ ions; on the other hand, smaller Yb^3+^ concentration grows larger Ag NCs and ET to Yb^3+^ ions occurs more efficiently at 410 nm excitation. Furthermore, the Ag NCs co-doping increases the Yb^3+^ lifetime at 980 nm, under 405 nm excitation, also corroborating the mentioned ET mechanism. A slight lifetime increase, from 0.83 to 0.87 ms, was observed when comparing GP 4.5Yb and GP 4.5Ag4.5Yb samples, respectively; on the other hand, comparing GP 2Yb and GP 4.5Ag2Yb samples, a larger growth occurs, from 0.79 to 0.94 ms. The reduction of Ag NCs lifetime and the Yb^3+^ ions lifetime growth confirms the ET from Ag NCs to Yb^3+^ ions under 405 nm excitation. Although the largest decrease for Ag NCs lifetimes was demonstrated for the GP 4.5Ag4.5Yb sample, under 405 nm excitation, which would suggest higher ET for this sample, it is the GP 4.5Ag2Yb sample that displayed the highest Yb^3+^ ions lifetime enhancement, when excited at the same wavelength; this result indicates that the larger Ag NCs of the GP 4.5Ag2Yb sample, when compared to those of GP 4.5Ag4.5Yb, have more adequate sizes to provide efficient ET to Yb^3+^ ions, under 405 nm excitation. Therefore, the ET mechanism from Ag NCs to Yb^3+^ ions (^2^F_7/2_ → ^2^F_5/2_) was attributed to the S_1_→T_1_ decay (spin-forbidden electronic transition between singlet–triplet states) of Ag NCs. Transmission electron microscopy results proved the amorphous nature of Ag NCs and their growth restriction in the presence of Yb^3+^ ions. In the absence of Yb^3+^ ions, we observed Ag NCs with size in the range of 2.0 to 7. 5 nm; with the addition of Yb^3+^ ions, the Ag NCs size is in the range of 0.5 to 5.5 nm and decreases to 1.6 to 3.2 nm when the concentration of the Yb^3+^ ions is enhanced. The present work shows not only the capability of hosting Ag NCs in GeO_2_-PbO glasses in the presence of Yb^3+^ ions but also the efficient ET mechanism between them. Moreover, it covers the lack in the literature as there are few reports that demonstrate this mechanism in other hosts, and in none of the cases GeO_2_-PbO glass is used, as normally the studies are performed with oxyfluorides. The results reported here, shown for the first time, suggest possible applications in the VIS to NIR regions, such as tunable CW laser sources and materials based on frequency down-conversion process to optimize photovoltaic performance.

## Figures and Tables

**Table 1:** PL decay lifetimes (fast and slow components) of Ag NCs: GP 4.5Ag sample under 405 nm excitation, and detection at 500, 550, and 600 nm.

Signal Wavelength (nm)	τ_fast_ (μs)	τ_slow_ (μs)
500	9.2	33
550	16.1	93.4
600	18.0	113.4

**Table 2:** PL decay lifetimes (fast and slow components) of Ag NCs: GP 4.5Ag, GP 4.5Ag2Yb, and GP 4.5Ag4.5Yb samples, under 355 nm excitation and detection at 550 nm.

Sample	τ_fast_ (μs)	τ_slow_ (μs)
GP 4.5Ag	12.3	75
GP 4.5Ag2Yb	7.0	31
GP 4.5Ag4.5Yb	2.0	12

**Table 3:** PL decay lifetimes (fast and slow components) of Ag NCs: GP 4.5Ag, GP 4.5Ag2Yb, and GP 4.5Ag4.5Yb samples, under 405 nm excitation and detection at 550 nm.

Sample	τ_fast_ (μs)	τ_slow_ (μs)
GP 4.5Ag	16.1	93.4
GP 4.5Ag2Yb	11.4	70
GP 4.5Ag4.5Yb	9.4	42

**Table 4:** PL decay lifetimes for GP 2Yb, GP 4.5Yb, GP 4.5Ag2Yb, and GP 4.5Ag4.5Yb samples under 405 nm excitation with detection at 980 nm.

Sample	Time (ms)
GP 2Yb	0.79
GP 4.5Yb	0.83
GP 4.5Ag2Yb	0.94
GP 4.5Ag4.5Yb	0.87

## Data Availability

The data presented in this study are available upon request from the corresponding author.
